# Development of an Advanced Sham Coil for Transcranial Magnetic Stimulation and Examination of Its Specifications

**DOI:** 10.3390/jpm11111058

**Published:** 2021-10-21

**Authors:** Mayuko Takano, Jiri Havlicek, Dan Phillips, Shinichiro Nakajima, Masaru Mimura, Yoshihiro Noda

**Affiliations:** 1 Department of Neuropsychiatry, Keio University School of Medicine, Tokyo 160-8582, Japan; mayuko.takano.31@keio.jp (M.T.); shinichiro.l.nakajima@gmail.com (S.N.); mimura@a7.keio.jp (M.M.); 2 TEIJIN PHARMA LIMITED, Tokyo 191-8512, Japan; 3 DEYMED Diagnostic s.r.o., 54931 Hronov, Czech Republic; jiri@deymed.com; 4 Brainbox Ltd., Cardiff CF10 1AF, UK; dan@brainbox-neuro.com

**Keywords:** sham coil, transcranial magnetic stimulation (TMS), magnetic flux, monophasic stimulation, figure-of-eight coil

## Abstract

Transcranial magnetic stimulation (TMS) neurophysiology has been widely applied worldwide, but it is often contaminated by confounders other than cortical stimulus-evoked activities. Although advanced sham coils that elaborately mimic active stimulation have recently been developed, their performance is not examined in detail. Developing such sham coils is crucial to improve the accuracy of TMS neurophysiology. Herein, we examined the specifications of the sham coil by comparison with the active coil. The magnetic flux and click sound pressure changes were measured when the stimulus intensity was varied from 10% to 100% maximum stimulator output (MSO), and the changes in coil surface temperature over time with continuous stimulation at 50% MSO for each coil. The magnetic flux change at the center of the coil showed a peak of 12.51 (kT/s) for the active coil, whereas it was 0.41 (kT/s) for the sham coil. Although both coils showed a linear change in magnetic flux as the stimulus intensity increased, due to the difference in coil winding structure, the sham coil took less than half the time to overheat and had 5 dB louder coil click sounds than the active coil. The sham coil showed a sufficiently small flux change at the center of the coil, but the flux change from the periphery of the coil was comparable to that of the active coil. Future use of high-quality sham coil will extend our understanding of the TMS neurophysiology of the cortex at the stimulation site.

## 1. Introduction

In recent years, transcranial magnetic stimulation (TMS) has been widely used to study neurophysiology of the cerebral cortex due to its low invasiveness and its ability to stimulate neurons at arbitrary times. TMS can locally stimulate any cerebral cortex by generating eddy currents at the stimulation site by instantaneously changing the magnetic field with TMS coil. However, since TMS simultaneously induces not only targeted cortical neuronal activity, but also somatosensory evoked activity from scalp stimulation, auditory evoked activity from the clicking sound generated by the coil during stimulation, and biophysical effects from the range of magnetic field generation, these confounding factors need to be considered and controlled as much as possible in the TMS neurophysiology.

To address these confounding factors and to ensure the reliability and validity of the intended TMS measurement of genuine cortical-derived neuronal activity, TMS studies thus far have been conducted using sham (placebo) stimuli as a control [1,2,3,4,5,6]. The ideal sham stimulation condition is one that adds scalp stimulation and sound stimulation sensations which are as identical as possible to the stimulation of the active coil, generating comparable magnetic field changes around the stimulation target as well. One sham stimulation condition that has been widely used in TMS research so far is to tilt the coil 45–90 degrees from the original stimulation position relative to the scalp and have the edge of the coil touch the scalp [1,2]. Furthermore, one study also used a control condition by placing the stimulation coil on a body part away from the target site, such as the left scapula [3]. Such methods make it possible to reduce the magnetic field to the target site; however, they cannot mimic somatic stimulus sensations, including pain sensation and muscle contraction over the stimulated site, and there are clear differences in psychological and physical responses for subjects due to the obvious gap in the stimulation site.

Recently, a sham control study that takes into account the effects of auditory and somatosensory stimulation from the TMS coil on the brain has been conducted by reproducing the somatosensory stimulation with peripheral electrical stimulation to the scalp and placing an active coil with a box between the electrodes to mimic the vibrations and sounds generated by the coil [4,5,7]. However, in the case of sham stimulation using electrical stimulation, the stimulation itself is clearly different from that of TMS, and there is a major concern that electroencephalography (EEG) changes induced by scalp electrical stimulation are fundamentally different from the genuine TMS-evoked EEG activities. Furthermore, most conventional sham coils were not designed to consider the effects of the fluctuating magnetic field generated around the coil. In other words, the conventional sham coils so far were set to have no or almost no fluctuating magnetic field formed by the coil.

Thus, to overcome such problems and limitations of using conventional sham coils or sham stimulation conditions, Rocchi et al. recently have investigated the neurophysiological effects of auditory (i.e., auditory evoked potentials) and somatosensory (i.e., somatosensory evoked potentials) stimulation generated by TMS on the motor cortex using a new type of sham coil (70 mm alpha sham coil, Magstim, Whitland, UK), which generates only weak magnetic fields from the stimulation coil [6]. However, to date, the engineering performance of the same type of sham coil has not been reported in detail so far.

Here, we aimed to examine the technical characteristics of a high-precision sham coil (DuoMAG 70BFP: recently developed by Deymed) by comparing it with an active coil (DuoMAG 70BF).

## 2. Materials and Methods

### 2.1. Structure of Active and Sham Coils and Characteristics of Monophasic Waveforms Induced by These Coils

In this study, we examined the technical characteristics of active and sham TMS coils with a 70 mm diameter figure-8 butterfly coil (DuoMAG 70BF, p/n: 47-620, SN: 47-6201402105E; DEYMED Diagnostic Ltd., Hronov, Czech Republic). Both coils look identical, but the internal structure of the coils is different, such that this sham coil can only effectively stimulate the peripheral area around the target site (DuoMAG 70BFP, p/n: 47-623, SN: 47-6230062106; DEYMED Diagnostic Ltd.). The magnetic stimulator used in this experiment was a DuoMAG MP (p/n: 47-000, SN: DM-3752103L).

**Figure 1:**
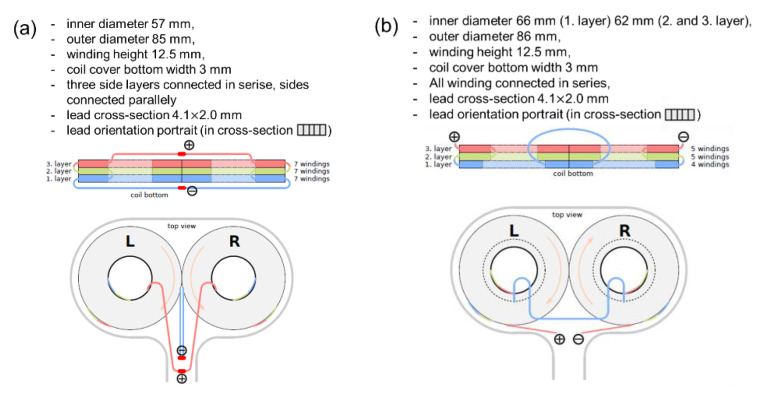
Structure of active and sham coils and their parameters. (**a**) represents an active coil (DuoMAG 70BF) and (**b**) shows a special sham coil (DuoMAG 70BFP). The top row is a cross-sectional view of the coil from the side, while the bottom row is a plain view from the top. The red, green, and blue colored areas and lines in the cross-sectional view indicate the coil windings and their layers. The doughnut-shaped white area inside of the coil indicates the inner empty core of the coil winding. The plus and minus in the figure represent the direction of the current flow in the coil along with the orange arrows drawn inside the left and right coils on the top view.

The structure and various parameters of the active and sham coils used in this experiment are shown in Figure 1. Both the active and sham coils consisted of three layers. The active coil layers on one side were connected in series with the layers on both sides connected in parallel, while the sham coil was all winding in series. More specifically, in the active coil, the coil was wound seven times in all three layers, whereas, in the sham coil, the first layer was wound four times and the second and third layers were wound five times. Therefore, the internal diameter of the active coil was 57 mm, while in the sham coil, the internal diameter of the first layer was 66 mm and that of the second and third layers was 62 mm.

There was no difference in the thickness of the coil windings between the active and sham coils. The active coil has a structure in which the current flows in the opposite direction, clockwise in the left winding and counterclockwise in the right winding, to enable local stimulation, as shown by the orange arrows in the top view of Figure 1. On the other hand, the sham coil was designed to stimulate the peripheral area around the target site and to offset central stimulation as much as possible, with the current flowing in the left and right windings in the same direction. Note that as for the waveform generated from the stimulator, we used a device that outputs a monophasic waveform for both the active and sham coils.

### 2.2. Comparison of Magnetic Flux Changes for Each Coil

**Figure 2:**
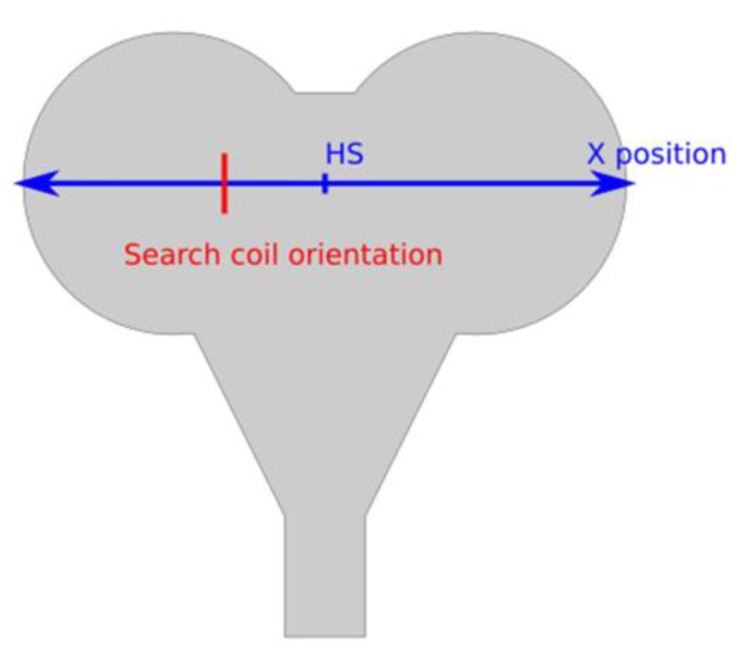
Schematic diagram of magnetic flux change measurement. The blue line indicates the position at the time of measurement, where the hot spot (HS) is 0, the right side shows positive and the left side shows negative. The red line shows the orientation of the search coil with respect to the coil to be measured.

We measured magnetic flux changes using a similar method as described in the paper by Smith et al., 2019 [8]. For the measurement of flux changes in the active and sham coils, we used a search coil made by Deymed (Hronov, Czech Republic; DuoMAG Search Coil; p/n: 47-664, SN: 47-6640012103). The schematic diagram of the measurement area is shown in Figure 2. The orientation of the search coil was perpendicular to *X*-axis, the center of *X*-axis was set to 0 mm, and the measurement step was set to 5 mm. The distance from the center of the search coil to the coil surface was 15 mm. The magnetic flux change rate was calculated using the following formula.

dB/dt(kT/s) = ((voltage on search coil(V)))/((search coil constant K (m^2^) × 100)), (1)

In addition, the magnetic flux change in each coil was measured when the maximum stimulator output (MSO) was varied from 10% to 100%. The search coil was positioned at a height of 15 mm from the coil surface for the measurements. The default value of 0.001183 (m^2^) was used for the constant K of the search coil. We also performed an independent *t*-test on the measured flux changes at the periphery and center of both coils at a significance level of α = 0.01.

### 2.3. Temperature Changes Associated with the Accumulation of TMS Pulses in Each Coil

The surface temperature of each coil was measured using a thermistor. This thermistor was first tested in a calibrated heated circulating bath (20 points in the temperature range of 25–45 °C) and later used for measuring the temperature of the coil. Stimulation conditions for both active and sham coils were set at 50% MSO for stimulation intensity and 0.5 Hz for stimulation frequency. The temperature at the start of the measurement was 25 °C until each coil overheated and automatically stopped.

### 2.4. Measurements of Coil Click Sound Pressure with Change of Stimulus Intensity

A commercially available digital sound level meter (Benetech, Kalisz, Poland; GM1356 s/n: JD: 2055939) was used to measure the coil sound pressure. To mimic the sound during head stimulation, the tip of the microphone was placed 10 mm from the left end of the coil, horizontal to the coil plane. The coil sound pressure was measured at 10% intervals between 10–90% MSO. For each stimulus intensity, the maximum values during five single-pulse stimuli were measured for 10 samples and averaged to obtain the coil sound pressure of the stimulus intensity at the time of measurement. The differences in the sound pressure of coil clicks generated by the active coil and sham coil at each stimulus intensity were compared with an independent *t*-test. The significance level α was set at 0.05/9 ≃ 0.0056.

## 3. Results

### 3.1. Monophasic Waveforms of the Active and Sham Coils

**Figure 3:**
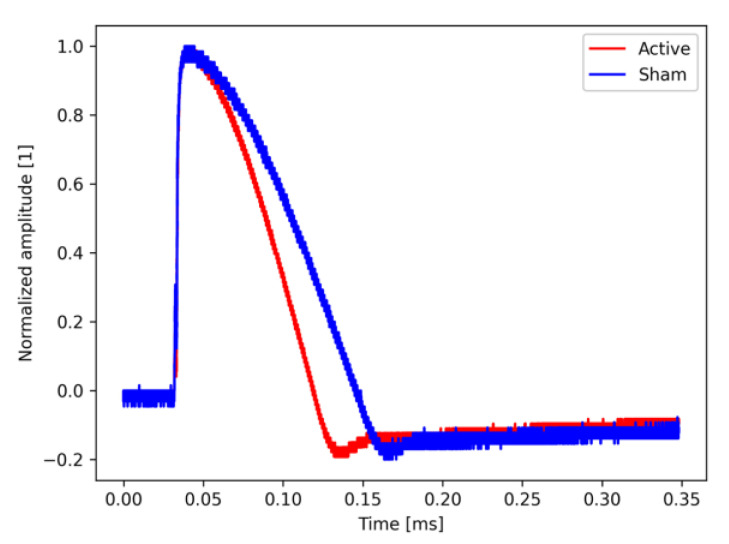
Monophasic waveform with active and sham coils. The horizontal axis shows the time and the vertical axis shows the amplitude normalized to 1 at the maximum value. The red line represents the active coil and the blue line indicates the sham coil.

The amplitude of the waveform has been normalized so that the maximum value is 1. The pulse width of the monophasic waveform by the active coil was approximately 0.10 ms, whereas that of the monophasic waveform by the sham coil was approximately 0.13 ms (Figure 3). The pulse width of the sham stimulation was set wider than that of the active stimulation, which was adjusted so that the stimulation sensation during head stimulation was as equivalent as possible between the active and sham stimulation.

### 3.2. Comparison of Magnetic Flux Changes in Each Coil

**Figure 4:**
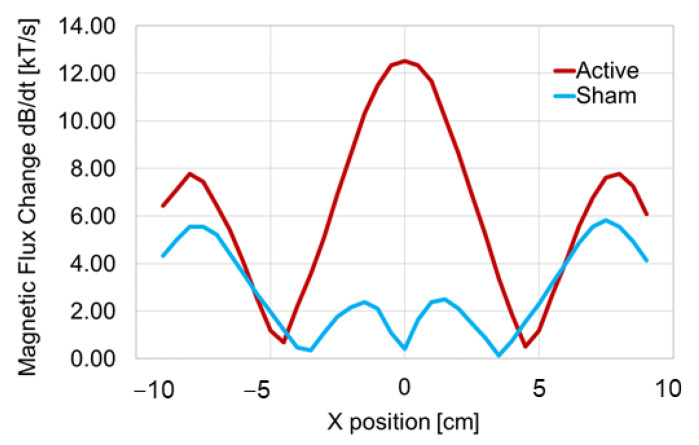
Schematic diagrams of the magnetic fields generated by the active and sham coils. The horizontal axis shows the position of the search coil in the coil to be measured and the vertical axis shows the magnetic flux change. The position of the search coil in the coil to be measured is shown on the horizontal axis, and the vertical axis shows the magnetic flux change. The center of the coil to be measured was set at X = 0 cm, and measurements were taken up to a position of 9 cm on either side. The red line represents the results of the active coil and the blue line shows the results of the sham coil. In the active coil, three peaks of magnetic flux change were observed, while in the sham coil, four peaks of magnetic flux change were observed.

The magnetic flux changes for each coil position using the search coil are shown in Figure 4. In the active coil, three peaks of magnetic flux change were observed. The left and right peaks of magnetic flux change were located approximately 8 cm away from the center of the coil, and the left and right peaks of magnetic flux change were approximately 7.78 (kT/s). The peak with the largest magnetic flux change occurred at the center of the coil, with 12.51 (kT/s). On the other hand, four peaks were generated from the sham coil. The left and right peaks appeared at a distance of approximately 8 cm from the coil center, as in the active coil, and the magnetic flux changes were 5.55 (kT/s) (left) and 5.82 (kT/s) (right), respectively. The two peaks close to the coil center showed magnetic flux changes of 2.37 (kT/s) at −1.5 cm and 2.50 (kT/s) at 1.5 cm, respectively. For the sham coil, the magnetic flux change at the center of the coil was 0.41 (kT/s).

**Figure 5:**
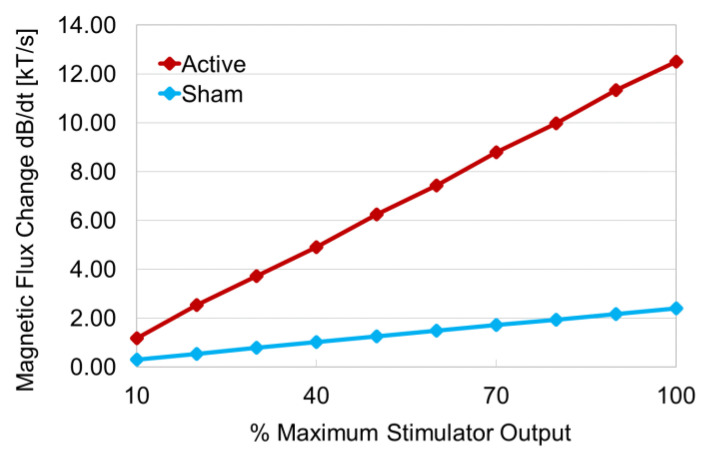
Relationship between magnetic flux and MSO strength. The horizontal axis shows the machine stimulator output (MSO) and the vertical axis indicates the magnetic flux change. MSO, which indicates the stimulus intensity of the TMS device, was increased from 10% to 100% in 10% intervals, and the magnetic flux change corresponding to each MSO for each coil was measured. The red line represents the measurement results of the active coil and the blue line shows the measurement results of the sham coil. In both coils, the magnetic flux change increased linearly with increasing stimulus intensity.

The magnetic flux changes in response to the stimulus intensity (MSO) of TMS were linear for each coil (Figure 5). For the active coil, the magnetic flux increased by approximately 1.26 (kT/s) for every 10% increase in MSO. On the other hand, for the sham coil, it increased by approximately 0.24 (kT/s) for every 10% increase in MSO. The magnetic flux changes at the minimum (10% MSO) and maximum (100% MSO) stimulus intensity of TMS in the experiment were 1.18 (kT/s) and 12.51 (kT/s) for the active coil, and 0.30 (kT/s) and 2.40 (kT/s) for the sham coil, respectively. The results for the periphery of the coils were as follows: left wing (−9~−5 cm): t_16_ = 1.27, *p* = 0.11; right wing (5~9 cm): t_16_ = 1.11, *p* = 0.14. On the other hand, the results for the center of the coils (−5~5 cm) were as follows: t_36_ = 5.79, *p* = 0.0000007. Thus, there was no significant difference in the flux change between the two coils at the periphery of both coils, but there was a significant difference in the flux change between the two coils at the center of the coil.

### 3.3. Coil Temperature Changes with Continuous Use of TMS

**Figure 6:**
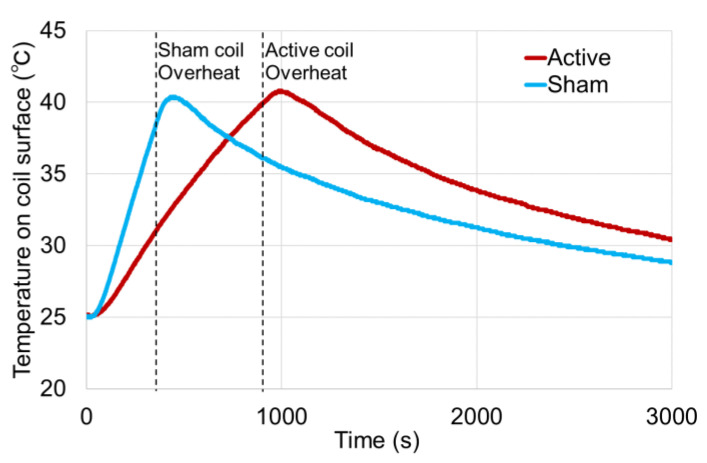
Relationship between coil surface temperature and time course of 0.5 Hz continuous stimulation in the active and sham coils. The horizontal axis indicates the time of measurement from the start of stimulation and the vertical axis shows the surface temperature of the coil. The stimulation was performed with 50% MSO, continuous single-pulse stimulation. The red line shows the temperature change of the active coil while the blue line indicates the temperature change of the sham coil. The dashed line shows the timing when each coil automatically stopped stimulation as a safety measure due to temperature increase.

The change in coil surface temperature with continuous TMS use at 0.5 Hz at a stimulation intensity of 50% MSO is shown in Figure 6. In the active coil, the coil temperature reached 40.2 °C at 923 s from the start of stimulation and overheated. On the other hand, in the sham coil, the coil temperature reached 38.4 °C at 353 s after the start of stimulation and overheated. Even after overheating, the surface temperature of each coil increased to nearly 40.3 °C. As a safety margin, this TMS coil is set to automatically stop the output from the stimulator so the surface temperature will reach a maximum of approximately 41.0 °C.

The increase in the coil surface temperature after the start of stimulation was almost linear in both active and sham coils, but the slope of the temperature increase in the sham coil was steeper than that in the active coil.

### 3.4. Comparison of the Click Sound Pressure Generated by the Active and Sham Coils during Stimulation

**Figure 7:**
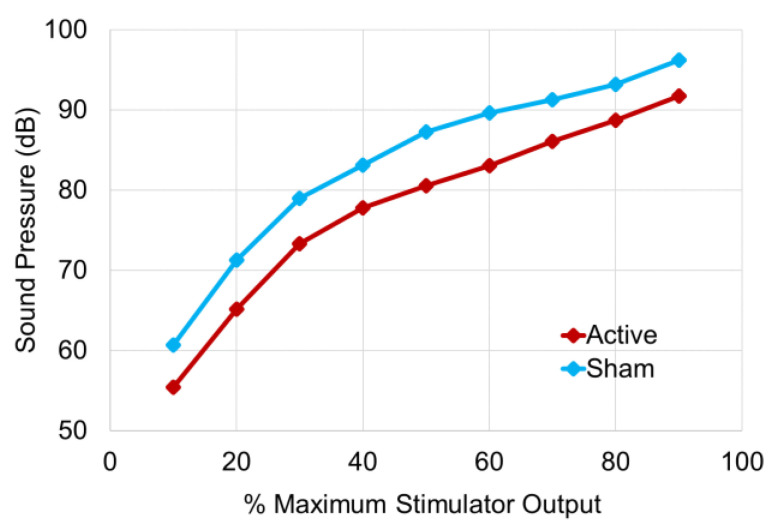
TMS click sound pressure during stimulation in the active and sham coils. The horizontal axis shows the stimulus intensity (MSO) and the vertical axis indicates the sound pressure. MSO was increased from 10% to 90% at 10% intervals, and the sound pressure corresponding to each stimulus intensity was measured for each coil. The red line shows the sound pressure of the click sound in the active coil while the blue line indicates the sound pressure in the sham coil. Each plot shows the average value of five measurements of the maximum value of five single stimulations.

The sound pressure values of the coil click sound generated from each coil when the MSO was varied from 10% to 90% are shown in Figure 7. For the active coil, the sound pressure ranged from 55.4 dB to 91.7 dB, while for the sham coil, the sound pressure ranged from 60.7 dB to 96.2 dB. At all stimulus intensities, the click sound of the sham coil was approximately 5 dB higher than that of the active coil. The changes in sound pressure against stimulus intensity for both coils were parallel and showed a similar pattern of sound pressure change. The results of the statistical analysis on the difference in sound pressure of the click sound generated from both coils were as follows: 10% MSO: t_18_ = −19.5, *p* = 7.14 × 10^−14^; 20% MSO: t_18_ = −110.4, *p* = 3.05 × 10^−27^; 30% MSO: t_18_ = −17.4, *p* = 5.23 × 10^−13^; 40% MSO: t_18_ = −105.1, *p* = 7.39 × 10^−27^; 50% MSO: t_18_ = −92.0, *p* = 8.08 × 10^−26^; 60% MSO: t^18^ = −24.0, *p* = 2.02 × 10^−15^; 70% MSO: t_18_ = −55.1, *p* = 8.04 × 10^−22^; 80% MSO: t_18_ = −29.9, *p* = 4.35 × 10^−17^; 90% MSO: t_18_ = −33.9, *p* = 4.66 × 10^−18^, indicating a significant difference in sound pressure between the active and sham coils at all measurement points.

## 4. Discussion

### 4.1. Characteristics of Sham Coil

With regard to the coil design (Figure 1), the active coil was designed to have the current flow clockwise in the left coil and counterclockwise in the right coil, while the sham coil was designed to have the current flow clockwise in both coils. By such a coil design, the active coil shows the maximum magnetic flux at the point where the left and right coils intersect, while the sham coil generates magnetic flux in the opposite direction from each coil at the intersection point of the left and right coils, and these magnetic fluxes cancel each other out. Therefore, in the sham coil, magnetic stimulation from the center of the coil does not fire the cerebral cortex directly under the same area, but only provides stimulation sensation similar to the active stimulation. For the monophasic waveform, the pulse width of the sham stimulation was about 0.03 ms longer than that of the active stimulation (Figure 3). This was attributable to the result of adjusting the waveform pulse width so that the intensity and sensation of the stimulation subjectively perceived by human subjects were identical when both coils were placed on the head under the same conditions and stimulated with the same stimulation intensity.

The magnetic flux change of the sham coil at X = 0 cm, which is the intersection point of the left and right coils, was limited to approximately one-twelfth that of the active coil. The relationship between the stimulus intensity and the magnetic flux change shown in Figure 4 demonstrated that for the sham coil, the magnetic flux change was 1.25 (kT/s) at 50% MSO and 2.40 (kT/s) at 100% MSO, which was approximately 1.9 times higher. Since the relationship between the stimulus intensity and the magnetic flux change was almost linear, the flux change in the *X*-axis also changed linearly, and the flux change at 100% MSO was 0.41 × 1.9 = 0.779 (kT/s). In the case of the sham coil, the flux change was sufficiently small even at 100% MSO. The magnetic flux change from 10% to 90% MSO in the sham coil was lower than the flux change at 10% MSO in the active coil, and the flux change at 100% MSO in the sham coil was also less than the flux change at 20% MSO in the active coil. On the other hand, in the sham coil, the peak of the magnetic flux was observed at X = −2 cm and 2 cm. Furthermore, at X = −8 cm and 8 cm, the second height peaks of the magnetic flux change were generated for both the active and sham coils, and the scalp stimulation around the target area was biophysically designed to give almost the same stimulation sensation regardless of a sham coil or an active coil. The temperature increase in the coil up to overheating by the same stimulus intensity was about 2.5 times faster in the sham coil than in the active coil (Figure 6). This is considered to be due to the difference in the structure of the coils. Note that the coil used in this study was not equipped with a cooling system.

Regarding the click sound pressures from the TMS coils during stimulation, the sham coil was approximately 5 dB louder than the active coil at all intensities. This was probably due to the difference in monophasic pulse width as well as the winding structure of the coils. In both coils, the slope of the increase in sound pressure from 10% to 30% MSO was steep, while the slope of the increase in sound pressure from 30% to 90% MSO was mild, indicating a non-linear change. However, the slope of the tangent line was almost the same for both coils.

### 4.2. TMS-EEG Study Using Sham Condition

The method of tilting the active coil by 90 degrees does not provide any stimulating sensation to the scalp, thus it remains unclear whether it truly meets the sham condition. The method in which active stimulation is performed away from the scalp at the target site may cause unexpected responses originating from non-target sites (whether it be brain or shoulder stimulation), and the gap in the setting between the active and sham conditions is too large because the coil for the sham condition is not touching the target site in the first place. There is a method to induce an auditory stimulus response by generating a sound that mimics a coil click sound in the target area of the head, but subjects are likely to suspect that the stimulation would be fake because the actual mimicking sound and the stimulus sensation on the scalp are somehow different from those during active stimulation.

In the method of applying electrical stimulation with a pair of surface electrodes attached directly under the center of the TMS coil to mimic the pain sensation caused by scalp muscle contraction with TMS, the current flows through the scalp, muscles, and cerebrospinal fluid to produce some form of electric field (E-field) in the brain, but the nature of this E-field is originally different from that of the E-field formed by TMS pulses [9]. In other words, in this sham stimulation condition, not only are the E-fields generated different between conditions, but also the electromagnetism effects of those E-fields on the brain would be different.

### 4.3. Solutions to the above Problems and Limitations with the New Sham Coil

Scalp sensation: The internal structure of the sham coil examined in this study was different from that of the active stimulation coil, but the external structure was identical. Thus, the sensation when the coil was placed on the head was the same as that of the active stimulation coil. In addition, although there was a large difference in magnetic flux change and induced magnetic field between the two coils, the magnetic field induced around the target area by the sham coil was the same as that in the active coil. Thus, the sensation brought to the scalp by magnetic stimulation with the sham coil was so elaborate that it was subjectively indistinguishable. Therefore, the present sham coil was able to wipe out the various problems mentioned above associated with mimicking the sensation of scalp stimulation by direct electrical stimulation.

Differences in the local induced magnetic field at the stimulation target: In the case of active stimulation, the highest magnetic flux change was observed just below the center of the coil (Figure 4), and previous electromagnetic studies on figure-8 coils have shown that the induced current density was highest directly beneath the intersection of the figure-8 coils, which enables local brain stimulation [10]. On the other hand, the magnetic flux change at the intersection point of the sham coil was almost zero, in contrast to the active coil. Therefore, the current density induced under the intersection of the coils was almost zero, and local brain stimulation under the intersection was considered to be impossible. Therefore, when the sham coil is placed on the head, the subjective stimulation sensation to the scalp is similar to that of the active coil, but the brain stimulation just beneath the target coil intersection cannot be performed.

Induced magnetic field around the stimulation target: In the sham coil, as shown in Figure 4, small peaks of magnetic flux change occurred at X = −2, 2 cm. At the same coordinates, the magnetic flux change in the sham coil was small compared to the flux change in the active coil, but, at the very least, it was found to generate a weak magnetic field on the scalp. Therefore, it is thought that the magnetic field change can be induced around the target site in the sham coil as well, which can provide somatosensory stimulation sensation around the stimulation target without providing effective magnetic stimulation to the target site. In addition, the magnetic flux changes outside X = −4, 4 cm showed similar peaks in the active and sham coils, although the intensity was slightly different, indicating that a similar induced current would be generated in the scalp corresponding to the areas surrounding the coils.

TMS click sounds: This sham coil was also able to generate the click sound peculiar to magnetic stimulation, which is generated by the pulse current flowing in the coil, with a sound quality almost identical to that of the active coil. However, due to the difference in the winding structure of the coils, there was a limitation that the click sound generated by the sham coil was approximately 5 dB higher than the click sound generated by the active coil. Furthermore, since this sham coil can be attached to the same stimulation site on the scalp as the active coil, the sound propagated by bone conduction could be felt as well.

Potential psychological effects of the appearance of the sham coil: The visual appearance of the sham coil was indistinguishable from that of the active coil, allowing subjects to remain blind to its appearance. In principle, there would be no psychological placebo or nocebo effects due to the appearance of the coil.

### 4.4. Future Direction

The sham coil introduced in the present study is much more reliable than the conventional sham coils because the stimulated sensation would be almost indistinguishable from the active coil for subjects, and their appearance is indistinguishable for examiners. Thus, in the future, TMS neurophysiological experiments using such an elaborate sham coil system as a control to precisely and non-invasively evaluate more genuine TMS-evoked responses derived from neural tissues at the stimulation site will greatly contribute to the development of human cerebral physiology.

### 4.5. Limitations of the Present Sham Coil

It is expected that by applying a monophasic pulse waveform to the sham coil, which was designed to provide a similar TMS sensation by the active coil to the scalp, the stimulation sensation to human subjects would be similar to that of the active coil at the same intensity. However, this study did not evaluate the difference of stimulation sensation between sham and active coils under the same stimulation intensity in human subjects, and it is necessary to evaluate the difference of stimulation sensation between the two coil conditions in human subjects. It is also important to note that the TMS click sounds in the ideal sham condition are generated in the same way as in the active condition. However, based on the comparison test of the sound pressure from these coils, it was found that the sham coil was about 5 dB louder than the active coil.

In the present experiment, since we did not measure the frequency components of the click sound between the active and sham coils, we could not analyze the difference in the frequency characteristics of each click sound. Therefore, it is possible that the subtle differences in the click sound of the two coils may cause some differences in the neurophysiological response of the auditory system. In addition, since the coil click sound was measured at a constant distance from the coil in this experiment, the effect of bone conduction caused by placing the TMS coil directly on a subject’s head could not be evaluated. Since bone conduction of TMS click sound could also affect the auditory processing mechanism as a sensory stimulus, this may be an issue to be investigated in the future [11]. However, the physical vibration of the TMS coil and the effects of air and bone conduction of the TMS click sound have been shown to be best mitigated by placing a layer of foam between the coil and the head, along with noise-masking with white noise or adapted noise [12]. Therefore, at the moment, at least, these strategies need to be prepared to achieve a more elaborate sham stimulation condition.

## 5. Conclusions

In this study, we succeeded in developing and implementing a reasonable and practical sham coil to overcome the various problems of conventional sham coils such as scalp electrical stimulation as well as coil click sounds. In the future, we will be able to evaluate more genuine TMS-evoked responses derived from the cerebral cortex than ever at the stimulation site by performing TMS neurophysiological experiments using a high-quality sham coil as a control.

## Data Availability

The data presented in this study are available on request from the corresponding author or DEYMED Diagnostic s.r.o.
